# Fragmented QRS on 12-lead EKG is an independent predictor for myocardial scar: a cardiovascular magnetic resonance imaging study

**DOI:** 10.1186/1532-429X-15-S1-P192

**Published:** 2013-01-30

**Authors:** Tarinee Tangcharoen, Weerapan Wiwatworapan, Watcharee Praserkulchai, Sirin Apiyasawat, Sukij Yamwong, Piyamitr Sritara

**Affiliations:** 1 Internal Medicine, Ramathibodi hospital, Mahidol University, Bangkok, Thailand; 2 Internal Medicine, Maharaj Hospital, Nakhonratchasima, Thailand

## Background

Fragmented QRS (fQRS) is a common EKG findings in general population. It has emerged as the independent predictor for cardiac events and all-cause mortality in patients with known coronary artery disease. However, none of the study evaluated the role of fQRS in patients without Q-wave on EKG. We aimed to evaluate whether fQRS is the predictor of cardiac function and myocardial scar in patients without Q-wave using cardiac MRI as the imaging tool.

**Figure 1:**
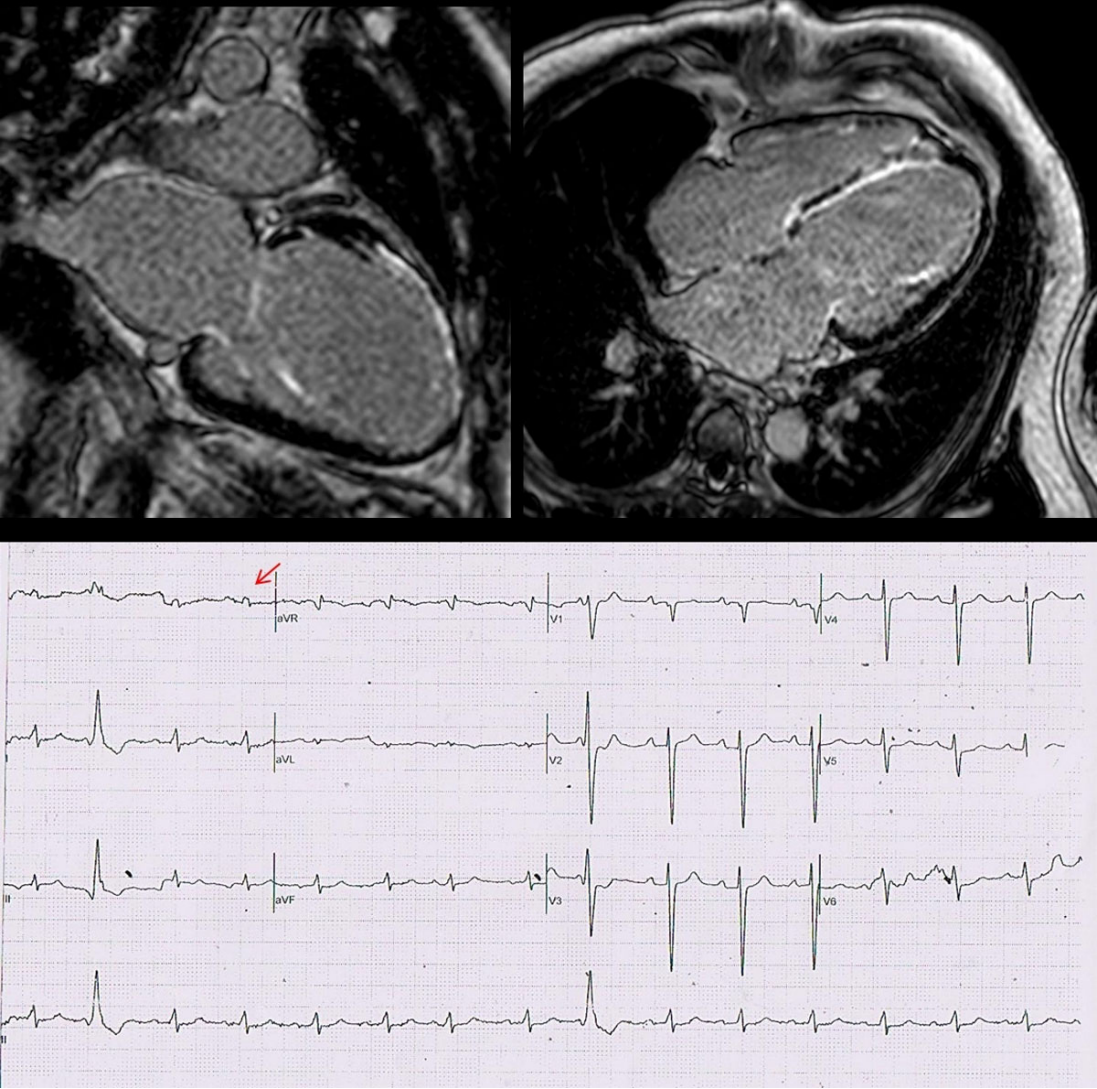
a 12-lead EKG of 56 year old man sent for cardiac MRI. Noted that no Q-wave was detected on his EKG despite of late gadolinium enhancement along septum and anterior wall. However, fragmented QRS was seen at lead I (arrow)

## Methods

250 patients who underwent stress cardiac MRI were included. The 12-lead EKG was analyzed by two independent observers. Fragmented QRS was defined as the presence of an additional R wave (R') or notching in the nadir of R or S wave, or the presence of more than 1 R'. Patients whom EKG showed Q-wave were excluded. Cardiac MRI was performed under 1.5T magnetic resonance scanner (Philips Achieva release 3.2). Resting left ventricular systolic function and mass was acquired with steady-state free precession sequence in short-axis view. All patients were given either Gadopentetate dimeglumine or Gadobenate demeglumine (total 0.2 mmol/kg) for late gadolinium enhancement sequence. Images were analyzed by 2 observers using Extended MR WorkSpace release 2.6. Impaired cardiac function was defined as left ventricular ejection fraction less than 50%. Myocardial scar was defined as hyper-enhanced area within left ventricular myocardium. Statistical analysis was done by SPSS.

## Results

Total 164 patients were finalized included (age, 63 + 11 years ; male, 42%; diabetes mellitus, 28.7%; hypertension, 64.6 % ; hyperlipidemia, 57.3%). Fragmented QRS was found in 49 patients (29.9%). Of 164 patients, 26 patients (15.9%) had myocardial scar whereas 23 patients (14%) had poor LV function. Although the LV mass, LVEDV and LVEF between the patients with fQRS and without fQRS was not significantly different, the patients with fQRS had more prevalence of myocardial scar than patients without fQRS (24.5% vs. 12.2%, p < 0.05). The sensitivity, specificity and negative predictive value of fQRS for detection of myocardial scar were 46 %, 73%, 88% respectively. Using multivariable regression analysis, the fQRS was a strong independent predictor for myocardial scar detection (OR 4.26, p = 0.026 ) after adjusted for other cardiovascular risk factors.

## Conclusions

Fragmented QRS is an independent factor for myocardial scar, therefore, patients with this abnormal EKG findings should have cardiac imaging investigation irrespective of conventional coronary risk factors or history of ischemic heart disease.

## Funding

None

**Table 1:** Multivariate regression analysis for myocardial scar detection

	Myocardial scar detected by late gadolinium enhancement MRI		
	OR	95% CI	p

age (years)	1.03	0.97 - 1.09	0.38
male gender	1.05	0.26 - 4.12	0.95
Diabetes	0.29	0.06 - 1.35	0.12
Hypertension	4.94	1.01- 24.23	0.049
Dyslipidemia	1.31	0.32 - 5.36	0.71
History of ischemic heart disease	0.63	0.11 - 3.72	0.61
fQRS	4.51	1.19 - 17.09	0.027
LV mass (gm)	1.01	1.001 - 1.026	0.034
LV ejection fraction (%)	0.89	0.85 - 0.94	0.001

